# Circulating microtranscriptome profiles reveal distinct expression of microRNAs in severe leptospirosis

**DOI:** 10.1371/journal.pntd.0008809

**Published:** 2020-11-11

**Authors:** Umaporn Limothai, Janejira Dinhuzen, Titipon Payongsri, Sasipha Tachaboon, Pisit Tangkijvanich, Natthaya Chuaypen, Nattachai Srisawat

**Affiliations:** 1 Center of Excellence in Hepatitis and Liver Cancer, Department of Biochemistry, Faculty of Medicine, Chulalongkorn University, Bangkok, Thailand; 2 Excellence Center for Critical Care Nephrology, King Chulalongkorn Memorial Hospital, Bangkok, Thailand; 3 Critical Care Nephrology Research Unit, Faculty of Medicine, Chulalongkorn University, Bangkok, Thailand; 4 Department of Anesthesiology, Faculty of Medicine, Chulalongkorn University, Bangkok, Thailand; 5 Division of Nephrology, Department of Medicine, Faculty of Medicine, Chulalongkorn University, and King Chulalongkorn Memorial Hospital, Bangkok, Thailand; 6 Academy of Science, Royal Society of Thailand, Bangkok, Thailand; 7 Tropical Medicine Cluster, Chulalongkorn University, Bangkok, Thailand; 8 Excellence Center for Critical Care Medicine, King Chulalongkorn Memorial Hospital, Bangkok, Thailand; Medical College of Wisconsin, UNITED STATES

## Abstract

Biomarkers to predict the severity of leptospirosis are still lacking. This study aimed to identify and validate microRNAs in patients with severe leptospirosis, that could potentially be used as biomarkers for predicting an unfavorable outcome. Serum samples were collected from participants with definite diagnosis of leptospirosis. The participants were divided into two groups, non-severe and severe leptospirosis, as defined by the Specific Organ Sequential Organ Failure (SOFA) Score of more than two in any organ. Microtranscriptome analysis was performed using the NanoString miRNA Expression Assay. The expression level of candidate miRNAs was then validated by quantitative RT-PCR. Based on the NanoString, the microtranscriptome profile of the severe group was significantly different from that of the non-severe group. Upregulation of miR155-5p, miR362-3p, miR502-5p, miR601, miR1323, and miR630 in the severe group were identified, and further investigated. A total of 119 participants were enrolled in the validation cohort. Serum miR155-5p and miR630 levels were significantly higher in the severe group compared to the non-severe group. The combined use of miR155-5p or miR-630 with serum bicarbonate levels had an AUC of 0.79 (95%CI; 0.69–0.89, p<0.001) in identifying the severity of the disease. This data provides the first evidence that the microtranscriptome profiles of patients with severe leptospirosis were different from the non-severe group. Serum miR155-5p and miR630 levels might be novel biomarkers for identifying severe leptospirosis.

## Author summary

Leptospirosis is a zoonotic disease of global importance, particularly in the tropics, including Thailand. The mortality rate could be as high as 40%. These patients often developed kidney failure, liver failure, or cardiopulmonary failure, and require a high level of care. Nevertheless, the mortality rate can be reduced to 5% if prompt intensive care is provided. Predicting those at risk of developing severe disease is challenging. However, biomarkers to predict severe leptospirosis are still lacking. Our data provided the first evidence that microtranscriptome profiles of severe leptospirosis were different from the non-severe group. This data provides the first evidence that the microtranscriptome profiles of patients with severe leptospirosis were different from the non-severe group. Serum miR155-5p and miR630 levels might be novel biomarkers for identifying severe leptospirosis.

## Introduction

Leptospirosis is a zoonotic disease of global importance, particularly in developing countries and tropical areas, and is caused by *Leptospira spp*., a genus of pathogenic, high-motile, obligate aerobic, Gram-negative spirochetes [1]. The median global incidence is approximately 5 cases per 100,000 population but cam be up to 95 cases per 100,000 population in some areas [2], with an average mortality of 0.8% [3]. In Thailand, the fairly recent (2003–2012) incidence of leptospirosis ranged from 5 to 10 per 100,000 population, with an approximately 1% mortality rate [4].

Nearly 30% of severe, leptospirosis infections are severe and these patients usually require intensive care unit (ICU) admission [5–8], with a mortality rate as high as 40% [6]. These patients often developed kidney failure, liver failure, or cardiopulmonary failure, and required high level of care. However, the mortality rate can be reduced to 5% if prompt intensive cares are provided [9]. This emphasized the importance of early recognition and treatment. Unfortunately, biomarkers to predict severe leptospirosis are still lacking.

Pathogen-, as well as host-related factors are believed to play important roles in the development of severe leptospirosis. When *Leptospira* bacteria invade the host, the immune system will be activated in order to annihilate the pathogens [10]. However, an inappropriate response may result in deleterious outcomes. An excessive inflammatory response arising from the loss of control of the immune system can cause extensive tissue and organ damage [11, 12], as seen in acute lung injury or alveolar hemorrhage [13].

MicroRNAs (miRNAs) are small non-coding RNAs of 20–22 nucleotides in length and have a major role in posttranscriptional regulation of gene expression. These small RNAs repress protein synthesis through base pairing with partially complementary sequences in the 3’ untranslated region of the targeted mRNAs, favoring their degradation, and so translational repression [14–16]. Each miRNA can bind to diverse-sequence mRNAs with varying degrees of complementarity, leading to the ability to control hundreds of genes simultaneously upon environmental changes [17]. Such fine-tune adaptation and response result in specific transcriptome profiles, for both genes and miRNAs. These defined expression patterns can theoretically be used as biomarkers, especially in handling the disease.

Moreover, miRNAs are associated with disease and can act as biomarkers or therapeutic targets [18, 19]. MiRNAs have been identified as potential biomarkers of infections caused by a range of pathogens including leptospirosis [20]. More recently, the emphasis has focused on the use of serum miRNAs as diagnostic and prognostic biomarkers of infectious diseases such as sepsis caused by multiple infectious agents [21–23] and viral hepatitis [24, 25]. For this reason, those miRNAs and their targets, that are modulated by the pathogen, could be essential markers to comprehend the pathophysiology of leptospirosis. This study aimed to identify the potential miRNAs associated with severe leptospirosis. Then, the candidate miRNAs were validated as biomarkers for severe leptospirosis.

## Methods

### Ethics statement

The study protocol was approved by the ethical committee, Faculty of Medicine, Chulalongkorn University, and the ethical committee for research in human subjects of Si Sa Ket Hospital (COA No.004 REC No. 071/2560). All participants gave written informed consent and the study was conducted according to the Helsinki Declaration and Good Clinical Practice guidelines.

### Study design and the patients

This study was multicenter prospective observational study at 15 hospitals in Sisaket province during February 2016 to May 2017. Patients suspicious of leptospirosis by physicians from initial clinical presentation who was admitted at each study site during December 1, 2017 to November 30, 2018 were asked for consents and subsequently included into study. Clinicians suspected the diagnosis of Leptospirosis based on typical syndrome stated in the classic WHO clinical criteria such as the presence of acute febrile illness (onset of fever less than 14 days), headache, myalgia with history of exposure to animal water reservoirs or flooded environments either at home or at work. Clinically suspected patients were defined as “Leptospirosis confirmed cases” if one of the following laboratory criteria were met: (i) identification of *Leptospira* from clinical specimen by 16S rRNA sequencing, (ii) *Leptospira* agglutination titer of ≥ 400 by the microscopic agglutination test (MAT) in one or more specimens, or a four-fold increase in the *Leptospira* agglutination titer between the acute and convalescent phases, and (iii) detection of pathogenic *Leptospira* DNA by PCR from a clinical specimen. Serum and plasma samples were stored in aliquots at -80°C until further analysis. In order to assess the severity of leptospirosis, the modified sequential organ failure assessment (mSOFA) score (which included coagulation, liver, cardiovascular, renal, and the need for mechanical ventilation support) were recorded. Severe leptospirosis was defined as leptospirosis confirmed patients with an organ specific mSOFA score of more than 2, while non-severe leptospirosis was defined as participants with organ specific mSOFA score equal to or less than 2.

### RNA isolation from serum samples

Total RNA was extracted from 200 μL of serum using the miRNeasy Serum/Plasma Kit (Qiagen, Gaithersburg, MD, USA) according to the manufacturer’s protocol. “3.5 μl of Spike-In Control (C. elegans miR-39 mimic, QIAGEN Cat. 219610) was added to each extraction as an internal control.” The RNA concentration and purity were evaluated using the DeNovix DS-11 Spectrophotometer (DeNovix Inc, Wilmington, DE).

### NanoString nCounter system assays

Three samples from non-severe group and nine samples from severe group were selected to determine the expression pattern of 800 human miRNAs using the nCounter Human v3 miRNA Expression panel employing the nCounter Analysis System (NanoString Technologies, Seattle, USA). This assay is designed to digitally quantify mature miRNAs. Each NanoString expression assay can run 12 samples simultaneously. Briefly, around 100 ng total RNA was preprocessed with Tags ligation followed by hybridization with Reporter CodeSet and Capture ProbeSet (nCounter Human v3 miRNA Expression Assay). Samples were processed using the NanoString Prep Station and transferred into the nCounter cartridge, which was placed into the nCounter Digital Analyzer to capture the image (280 fields of view) and data. The miRNA data analysis was performed using the nSolver software, and miRNA profiling data were normalized using the average signals obtained from the top 100 miRNAs.

### Validation of NanoString results by qRT-PCR analysis

From the NanoString analysis, candidate miRNAs that showed significant differential the expression between the non-severe group and severe group were selected. After filtering, six candidate miRNAs, (miR-502-5p, miR-601, miR-362-3p, miR-1323, miR-630 and miR-155-5p) with log_2_ fold-change ≥ 1.5 and p-value < 0.05, were selected to validate their circulating expression levels by qRT-PCR analysis. For qPCR validation, universal stem-loop RT-qPCR was used to detect mature miRNA molecules as previously described [26]. Briefly, the cDNA was synthesized from purified total RNA using the SL-poly (A) sequence(GTCGTATCCAGGCAGGGTCCGAGGTATTCGCACTGGATACGACAAAAAAAAAAAAAAAAAAVN) and RevertAid First Strand cDNA Synthesis Kit (Cat No. 1622, Thermo Scientific, USA). After quantitative real-time PCR was done (StepOne Plus Real-time PCR System (Applied Biosystems, USA)), the SYBR Green system (QPCR Green Master Mix HRox, Cat No. BR0500402, Biotech rabbit, Germany) was used to assess the level of candidate miRNAs as described previously [26]. Sequences of the primers used in this study are as follows: miR-502-5p:5’ATCCTTGCTAT CTGGGTGCTA-3’ (forward), miR-601: 5’-TGGTCTAGGATTGTTGGAGGAG-3’ (forward), miR-362-3p:5’-AACACACCTATTCAAGGATTCAA-3’ (forward), miR-1323: 5’ TCAAAACTGAGGGGCATTTTCT-3’ (forward), miR-630:5’-AGTATTCTGTACCAGGGAAGGTAA-3’ (forward), miR-155-5p:5’-TAATGCTAATCGTGATAGGGGTT-3’ (forward), miR-16-5p:5’CAGCACGTAAATATTGGCG-3’ (forward) and SL-polyA-R:5’GCAGGGTCCGAGGTATTCG-3’ (universal reverse). All qRT-PCR reactions were performed in duplicate and the miRNA expression level was normalized to the internal control (hsa-miR16-5p), with the relative expression levels calculated by the 2^-ΔΔCT^ method.

### Plasma IL-6 measurement

Interleukin-6 was measured using human IL-6 immunoassay (Quantikine QuicKit ELISA by R&D Systems, Minneapolis, Minn., USA) according to manufacturer protocol.

### Aims (outcomes) and statistical analysis

The primary aim (outcome) was to determine the association between serum miRNAs and severity, and mortality after enrollment of patients with leptospirosis. The secondary aims were to assess the prognostic ability of serum miRNAs and clinical parameters. We also added miRNA into clinical predictor scores to assess the additive prediction ability of combined clinical predictor scores and serum miRNAs.

The NanoString analysis was performed using the nSolver Analysis Software (Version 4.0). Continuous variables are presented as the mean ± one standard deviation (SD) in case of a normally distribution, and as a median and interquartile range (IQR) in case of non-normally distributed variables. Student’s t test or Mann-Whitney test was used to analyze the differences between two continuous variables. Categorical variables were presented as numbers with percentages, and were compared using the Chi-square test. Spearman’s rho was used to express correlations between miRNAs and other clinical parameters.

The association of miR155-5p and miR630 with disease severity was calculated using individual univariate binary logistic regression analyses. In addition, we used a receiver-operating-characteristic (ROC) approach to determine the extent to which miR155-5p and miR630 predicts either the disease severity or the mortality in the patients with leptospirosis. A p-value of less than 0.05 was considered statistically significant. All statistical analyses were performed with SPSS Version 22 software (SPSS, Chicago, IL), and figures were drawn using GraphPad Prism 8 (GraphPad Software Inc., California, USA).

## Results

### Patient characteristics

A total of 119 leptospirosis patients met the inclusion criteria of this study, of which 82 were classified as non-severe and 37 were severe leptospirosis. The patient characteristics of the two groups are presented in Table 1. A total of 12 serum samples, in which three samples were obtained from non-severe patients and nine samples were obtained from severe patients, were used for microRNA transcriptomic analysis in the discovery phase. All 119 serum samples from leptospirosis patients were further validated. The demographic data including the age, sex, body weight, height, BMI, and days of fever, were not significantly different between the two groups The severe leptospirosis group had a higher mSOFA score (6 vs 1, p<0.001), mortality (21.6% vs 0%, p<0.001), longer length of hospital stay (6 days vs 3 days, p<0.001), more use of mechanical ventilation (37.8% vs 0%, p<0.001), and ICU admission (24.3% vs 0%).

**Table 1:** Baseline demographic data.

Clinical Characteristics	Discovery Set (Nanostring)		Validation set (RT-qPCR)		
	Non-severe (N = 3)	Severe (N = 9)	Non-severe (N = 82)	Severe (N = 37)	P-value
**Demographic data**					
Age; years (mean±SD)	57±3	51±19	52±17.41	51±1.649	0.810
Sex; male (N, %)	2 (66.7%)	9 (100.0%)	66(80.49%)	31 (83.78%)	0.668
Body weight; kg (median, IQR)	59.00 (57.65, 63.00)	54.50 (50.00, 61.00)	56.00 (48.50, 63.50)	57.00 (50.00, 61.00)	0.933
Height; cm (median, IQR)	165.00 (162.50, 167.50)	160.00 (158.00, 160.00)	162.00 (155.50, 167.00)	162.00 (158.00, 165.00)	0.476
BMI; kg/m2 (mean±SD)	22.34±2.12	22.08±3.06	21.85±3.44	22.15±3.74	0.279
Alcoholism (N, %)	0 (0.00%)	1.00 (11.10%)	3 (3.66%)	3 (8.11%)	0.312
Smoking (N, %)	0 (0.00%)	5 (55.56%)	37 (45.12%)	17 (45.90%)	0.672
Day of fever (median, IQR)	1 (1, 2)	3 (0, 5)	3 (1, 4)	2 (1, 4)	0.876
**Comorbid diseases**					
Diabetes mellitus (N, %)	1(33.30%)	0 (0.00%)	4 (4.88%)	0 (0.00%)	0.169
Hypertension (N, %)	1(33.30%)	0 (0.00%)	8 (9.76%)	3 (8.11%)	0.759
Dyslipidemia (N, %)	0 (0.00%)	0 (0.00%)	1 (1.22%)	0 (0.00%)	0.497
Ischemic heart disease (N, %)	0 (0.00%)	0 (0.00%)	0 (0.00%)	0 (0.00%)	NA
Peripheral vascular disease (N, %)	0 (0.00%)	0 (0.00%)	0 (0.00%)	0 (0.00%)	NA
Malignancy (N, %)	0 (0.00%)	0 (0.00%)	0 (0.00%)	0 (0.00%)	NA
Cerebrovascular disease (N, %)	0 (0.00%)	0 (0.00%)	0 (0.00%)	0 (0.00%)	NA
Chronic liver disease (N, %)	0 (0.00%)	0 (0.00%)	0 (0.00%)	2 (5.4%)	0.035*
Chronic kidney disease (N, %)	0 (0.00%)	0 (0.00%)	1 (1.22%)	0 (0.00%)	0.500
Pulmonary disease (N, %)	0 (0.00%)	0 (0.00%)	0 (0.00%)	0 (0.00%)	NA
**Physical examination**					
Body temperature; °C (mean±SD)	38.57±0.38	37.80±1.57	38.37±1.11	37.92±1.44	0.052
Systolic blood pressure; mmHg (mean±SD)	123.33±17.39	99.40±10.95	116.11±22.40	103.10±18.85	0.005*
Mean arterial pressure; mmHg (median, IQR)	86.33 (81.50, 98.00)	76.67 (70.67, 76.67)	80.00 (75.50, 87.67)	76.67 (70.67, 77.00)	0.013*
**Laboratory finding**					
Hemoglobin; g/dL (mean±SD)	11.57±1.33	11.24±3.35	12.21±1.78	12.31±6.03	0.270
WBC; cellsx10^3^μL (mean±SD)	12.47±4.93	16.88±4.95	9.95±3.64	10.72±5.74	0.577
Platelets; cellsx10^3^μL (median, IQR)	189.00 (188.50, 286.50)	63.00 (25.00, 69.00)	148.00 (98.00, 207.00)	37.00 (30.00, 69.00)	<0.001*
Creatinine; mg/dL (median, IQR)	0.97 (0.89, 1.01)	4.56 (2.90, 7.51)	1.06 (0.92, 1.30)	3.50 (1.03, 4.70)	<0.001*
Glomerular filtration rate; mL/min (mean±SD)	82.61±5.84	23.382±22.50	77.41±25.90	42.24±37.06	<0.001*
HCO3-; mmol/L (mean±SD)	26.97±2.00	18.12±5.78	25.83±3.15	20.45±5.99	<0.001*
Total bilirubin; g/dL (median, IQR)	0.70 (0.50, 0.85)	1.60 (1.10, 8.30)	1.00 (0.70, 1.85)	1.90 (1.50, 4.80)	<0.001*
Direct bilirubin; g/dL (median, IQR)	0.40 (0.30, 0.65)	1.50 (0.90, 7.00)	0.58 (0.20, 0.85)	1.10 (0.70, 3.40)	<0.001*
SGOT; U/L (median, IQR)	76.00 (55.00, 130.50)	86.00 (39.00, 98.00)	48.00 (35.00, 87.50)	98.00 (39.00, 170.00)	0.052
SGPT; U/L (median, IQR)	75.00 (52.00, 143.00)	60.00 (36.00, 61.00)	41.00 (25.00, 80.50)	60.00 (32.00, 125.00)	0.636
Albumin; g/dL (mean±SD)	3.30±0.36	3.24±0.77	3.68±0.51	3.12±0.77	0.119
F. qPCR LipL32 (median, IQR)	188.00 (184.00, 282.72)	270.66 (192.76, 1269.82)	377.44 (121.21, 1722.64)	768.29 (192.76, 5951.83)	0.105
Positive hemoculture (N, %)	0 (0.00%)	0 (0.00%)	6 (7.32%)	2 (5.41%)	0.716
**Outcome**					
SOFA score (median, IQR)	0 (0.00%)	8 (5, 10)	1 (1, 3)	6 (5, 8)	<0.001*
Death (N, %)	0 (0.00%)	0 (0.00%)	0 (0.00%)	8 (21.62%)	<0.001*
Length of hospital stay; days (median, IQR)	3 (3, 4)	9 (6, 11)	3 (2, 4)	6 (5, 13)	<0.001*
Mechanical ventilation (N, %)	0 (0.00%)	3.00 (33.30)	0 (0.00%)	14 (37.84%)	<0.001*
ICU admission (N, %)	0 (0.00%)	5.00 (55.60)	0 (0.00%)	9.00 (24.30%)	<0.001*

WBC: white blood cell, SGOT: serum glutamic oxaloacetic transaminase, SGPT: serum glutamic, pyruvic transaminase, HCO: bicarbonate. Continuous data were expressed as means ± standard deviation (SD) or median and interquartile range (IQR). Categorical variables were expressed as numbers (%)

*, P-value<0.05, NA; not available

### Expression profiling of microRNAs in serum

**Fig 1:**
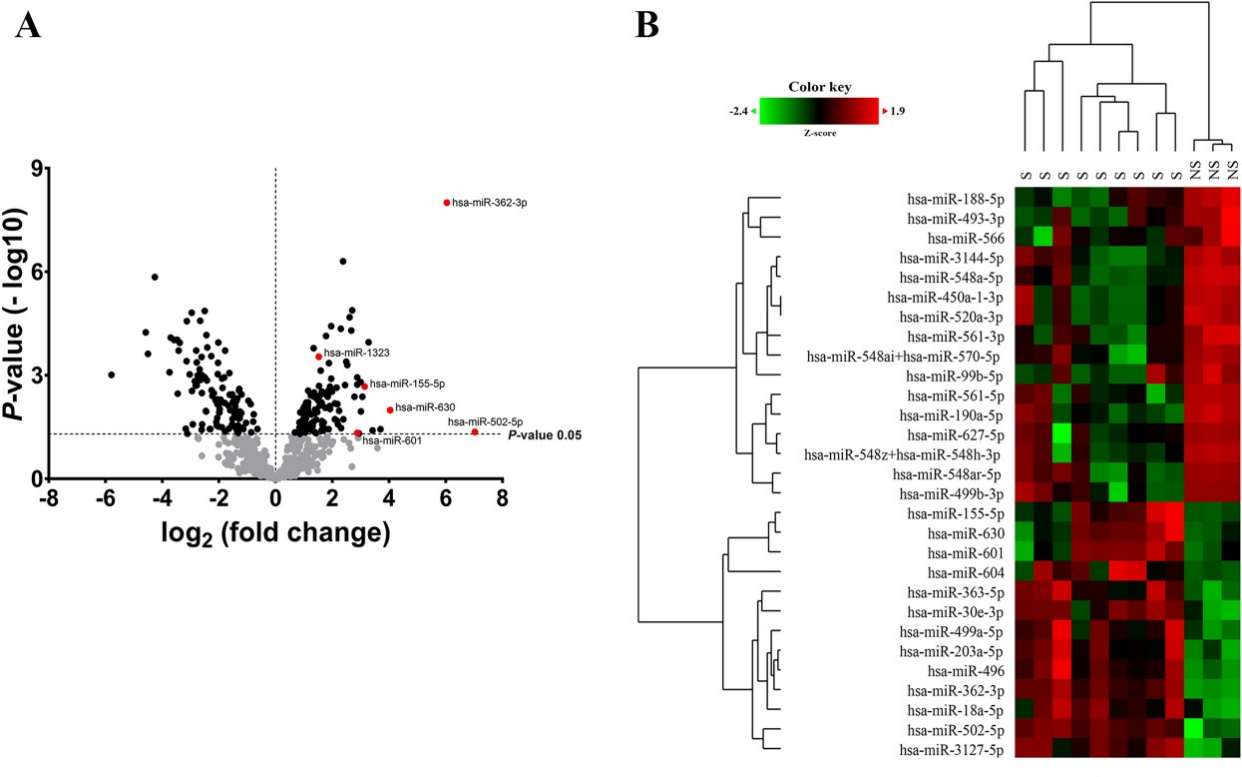
Volcano plot and heatmap for differentially expressed miRNAs between severe and non-severe leptospirosis groups. (A) Volcano plot for differentially expressed miRNAs, showing distribution of significance [-log10(p-value)] vs. fold change [log2(fold change)] for all genes. The miRNAs selected for qRT-PCR validation are highlighted with red dots and gene symbols marked (Fold change ≥1.5, p-value <0.05). The dots in black are significant differentially expressed miRNAs (p ≤ 0.05). (B) Heat map of significant differentially expressed miRNAs. Heatmap shows the signal of 29 miRNAs (p-value <0.05; log2fold change ±3).

Based on the NanoString platform, expression levels of 800 microRNAs were obtained. A volcano plot (Fig 1A) demonstrated that 231 miRNAs significantly differentially expressed between the non-severe group and the severe group (p<0.05). Raw data and normalized data outputs from nSolver 4.0 Analysis Software was available in S1 File. The heatmap and hierarchical analysis of 29 miRNAs are shown in Fig 1B. An average signal (log2) of the samples were hierarchically clustered. We observed a clustering of samples based on the severity of the disease, with the severe group clustered closer together and clearly separated from the non-severe group (Fig 1B). After filtering, six candidate miRNAs (represented by red points in Fig 1A), including miR-502-5p, miR-601, miR-362-3p, miR-1323, miR-630 and miR-155-5p in the serum specimens, were selected for further validation in serum specimens by qRT-PCR analysis.

### Validation of miRNA ratios by qRT-PCR

**Fig 2:**
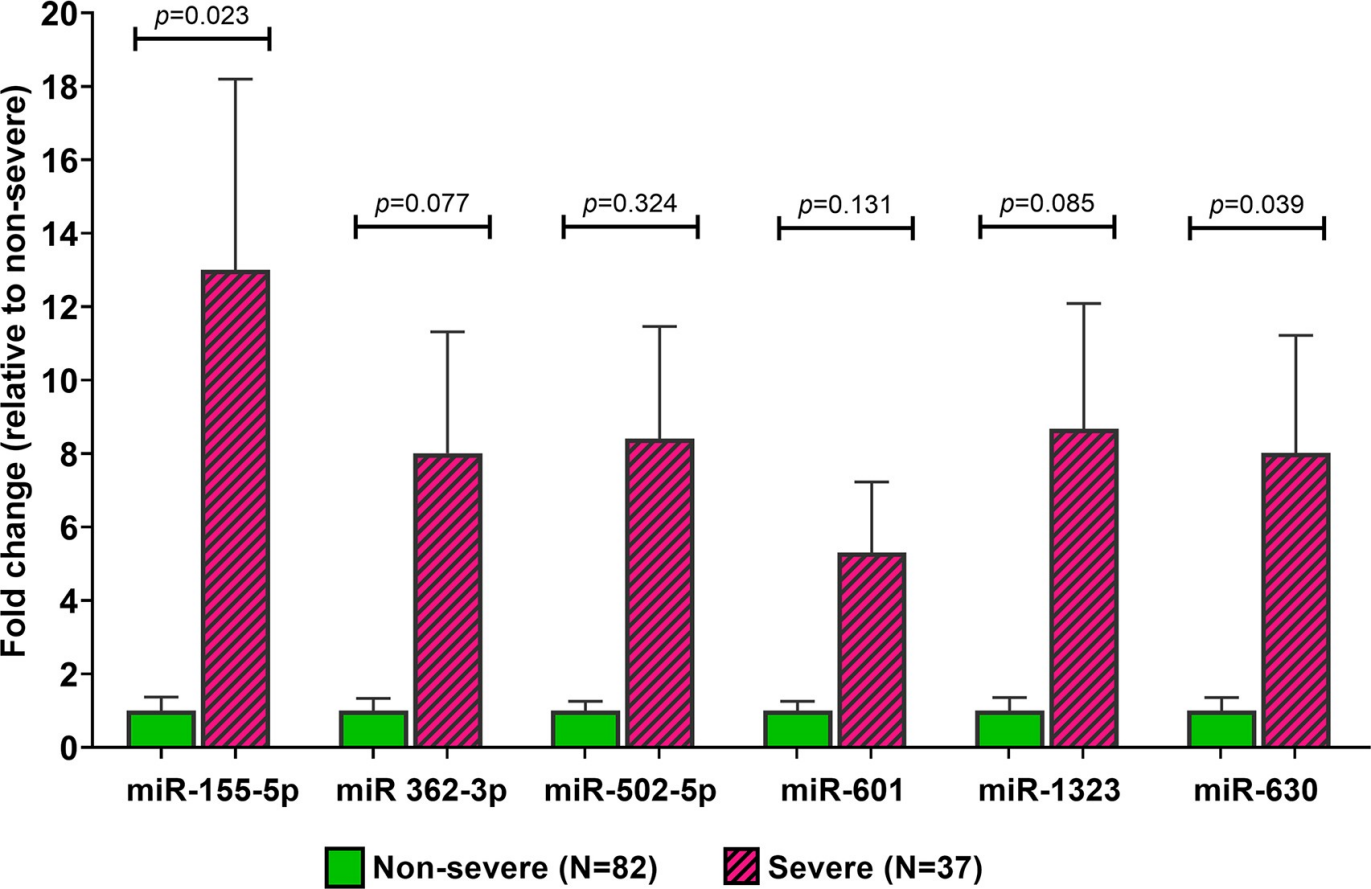
Relative expression of circulating microRNAs in patients with leptospirosis.

A total of 119 serum samples from the leptospirosis-positive patients were further investigated for their expression levels of the six selected miRNA, with the relative expression level of each miRNA were shown in Fig 2. The circulating miR-155-5p level in the severe leptospirosis group was significantly higher than in the non-severe group (13.00 ± 31.61 vs. 1.00±3.35, p = 0.023), as were the miR-630 levels (8.02±19.44 vs 1.00±3.23, p = 0.039). In contrast, there was no significant difference in the circulating levels of miR-502-5p (8.41±18.55 vs. 1.00±2.30, p = 0.321), miR-601 (5.31±11.68 vs. 1.00±2.28, p = 0.130), miR-362-3p (8.01±20.12 vs. 1.00±3.01, p = 0.076), and miR-1323 (8.67±20.76 vs. 1.00±3.22, p = 0.084). In further analysis, we compared the differential expression of serum miRNA levels in patients with leptospirosis to healthy donors. Our result showed that miR155-p and miR630 expression levels in the severe leptospirosis group were significantly higher than healthy donor group (S1 Fig).

### Correlations of serum miRNA levels, disease severity and markers of inflammation

The result showed that total mSOFA score had a positive correlation with both miR155-5p (r = 0.236, p = 0.010), and miR630 (r = 0.236, p = 0.010). Also among patients with severe leptospirosis, total mSOFA were correlated with both miR155-5p (r = 0.493, p = 0.002) and miR630 (r = 0.508, p = 0.001). The relationship between serum miRNAs levels and length of hospital stay was also examined. Among patients with severe leptospirosis, both miR155-5p (r = 0.521, p = 0.005) and miR630 (r = 0.522, p = 0.005) was correlated with length of hospital stay.

In terms of determining correlations between serum miRNA levels and other markers of inflammation. Levels of IL-6 were measured in plasma from 32 leptospirosis participants (18 non-severe and 14 severe leptospirosis). The result showed that the IL-6 levels were correlated with both miR155-5p (r = 0.586, p<0.000) and miR630 (r = 0.559, p = 0.001).

### Serum miRNAs associated with severity and mortality of leptospirosis

**Fig 3:**
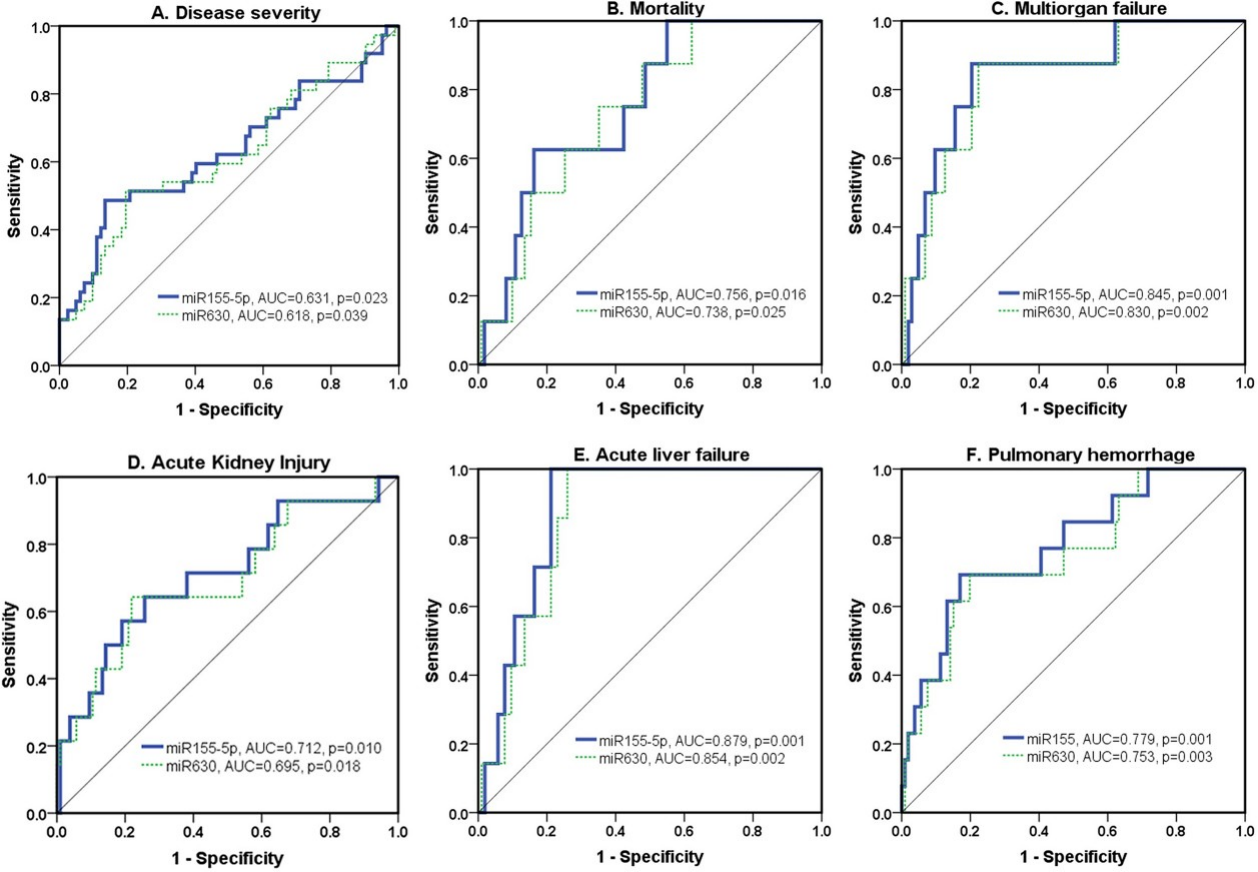
Receiver operating characteristic curve (ROC) for the association between serum miRNAs and (A) severity, (B) mortality, (C) multi-organ failure, (D) acute kidney injury, (E) acute liver failure, and (F) pulmonary hemorrhage.Next, a stepwise analysis combining the serum miRNAs levels with clinical predictors was performed to assess the discriminatory characteristics and build a most distinct severity predictor model. The addition of six serum miRNAs to the bicarbonate (HCO3-) level had the best discriminatory performance (combined AUCROC = 0.82, 95%CI; 0.73–0.92, p = <0.001) as shown in model 2 (Table 2).

The ROC curve for miR155-5p and miR630 were generated on the same graph to compare their diagnostic accuracies. As shown in Fig 3A, the area under the ROC curve (AUROC) of the severe leptospirosis groups and the non-severe groups was 0.63 (95% CI;0.513–0.748, p = 0.023) for miR155-5p and 0.62 (95%CI;0.50–0.73, p = 0.039) for miR630. Additionally, the AUROC for predicting the mortality of leptospirosis was 0.76 (95%CI;0.61–0.90, p = 0.016) for miR155-5p and 0.74 (95%CI,0.59–0.88, p = 0.025) for miR630 (Fig 3B).

**Table 2:** The area under the curve (AUC) for the association between serum miRNAs and severe leptospirosis.

No	Parameter	Model 1			Model 2		
		Parameter alone			Parameter +HCO3		
		AUC	(95%CI)	p-value	AUC	(95%CI)	p-value
1	miR155-5p	0.63	(0.51–0.75)	0.023*	0.79	(0.69–0.89)	<0.001*
2	miR362-3p	0.60	(0.48–0.72)	0.076	0.79	(0.69–0.89)	<0.001*
3	miR502-5p	0.56	(0.44–0.67)	0.321	0.79	(0.69–0.90)	<0.001*
4	miR601	0.59	(0.47–0.71)	0.130	0.80	(0.70–0.90)	<0.001*
5	miR1323	0.60	(0.48–0.72)	0.084	0.79	(0.69–0.89)	<0.001*
6	miR630	0.62	(0.50–0.73)	0.039*	0.79	(0.69–0.89)	<0.001*
7	Bicarbonate	0.78	(0.68–0.88)	<0.001*			
8	miR155+miR630	0.66	(0.55–0.77)	0.006*	0.79	(0.69–0.89)	<0.001*
9	Combined 6 miRNAs	0.67	(0.56–0.78)	0.003*	0.82	(0.73–0.92)	<0.001*

Severe forms of leptospirosis affect multiple organ systems. The ROC analysis was performed to evaluate the association between serum miRNAs and individual organ failures. For multiorgan failure, miR155-5p had the highest AUROC of 0.85 (95%CI; 0.71–0.98, p = 0.001) followed by miR630 with an AUROC of 0.83 (95%CI; 0.69–0.97, p = 0.002) (Fig 3C). Renal involvement is also notable in leptospirosis, where in this study the AUROC in predicting acute kidney injury (AKI) was 0.71 (95%CI; 0.56–0.87, p = 0.010) for miR155-5p and 0.70 (95%CI; 0.53–0.86, p = 0.018) for miR630 (Fig 3D). Similarly, the AUROC for predicting acute liver failure (ALF) was 0.88 (95%CI; 0.806–0.952, p = 0.001) for miR155-5p and 0.85 (95%CI, 0.77–0.94, p = 0.002) for miR630 (Fig 3E). Lastly, the predictive value of serum miRNAs for pulmonary hemorrhage was also significant for miR-155-5p (AUROC was 0.78; 95%CI; 0.643–0.914, p = 0.001) and miR630 (AUROC = 0.75, 95%CI; 0.61–0.90, p = 0.003) as shown in Fig 3F.

Table 3 lists the derived sensitivities and specificities for various cutoffs of each serum miRNAs to provide the maximum summation of the sensitivity and specificity. The miRNA155-5p at a cutoff of 4.9 (sensitivity of 0.51 and specificity of 0.79) and miR630 at a cutoff of 5.6 (sensitivity of 0.51 and specificity of 0.80) had a high combined sensitivity and specificity and so might act as biomarkers for the prognosis of both the severity (in an organ-specific manner) and mortality of leptospirosis.

**Table 3:** Serum miRNAs level at the best cut-off values for diagnosis severe leptospirosis.

Biomarkers	Cut-off	Sensitivity	Specificity
miR155-5p	1.2	0.62	0.54
	4.9	0.51	0.79
miR362-3p	1.7	0.62	0.46
	4.4	0.51	0.73
miR502-5p	1.6	0.62	0.49
	2.1	0.54	0.54
miR601	1.1	0.59	0.51
	4.2	0.54	0.73
miR1323	1.9	0.54	0.60
	6.4	0.46	0.79
miR630	1.8	0.59	0.54
	5.6	0.51	0.80

### Univariable and multivariable regression analysis

Based on the regression analysis, circulating miRNAs were further investigated to identify the variables associated with the severity of leptospirosis (Table 4). It was shown that high miR155-5p level was associated with the severity in the univariate model with an OR of 4.0 (95%CI, 1.75–9.32, p = 0.001). Moreover, the low miR155-5p was almost statistically significance with an adjusted OR of 2.69 (95%CI, 0.97–7.42, p = 0.057).

**Table 4:** Analysis of biomarkers associated with severe leptospirosis.

Variable	Model 1			Model 2*		
	Crude OR	(95%CI)	*p*-value	Adjusted OR	(95%CI)	*p*-value
miR155-5p (<4.9 vs ≥4.9)	4.04	(1.75–9.32)	0.001*	2.69	(0.97–7.42)	0.057
miR362-3p (<4.4 vs ≥4.4)	2.88	(1.28–6.46)	0.010*	1.57	(0.58–4.22)	0.372
miR502-5p (<1.6 vs ≥1.6)	1.57	(0.71–3.46)	0.269	1.24	(0.49–3.15)	0.659
miR601 (<4.2 vs ≥4.2)	3.21	(1.43–7.22)	0.005*	1.93	(0.73–5.12)	0.188
miR1323 (<6.4 vs ≥6.4)	3.25	(1.41–7.52)	0.006*	1.88	(0.67–5.29)	0.229
miR630 (<5.6 vs ≥5.6)	4.35	(1.8710.14)	0.001*	2.37	(0.85–6.61)	0.098

Data were expressed as odds ratio (OR) and 95% confidence intervals (CI)

*, *P-*value < 0.05

Adjusted for serum bicarbonate level

## Discussion

In our study, miRNA transcriptomic analysis demonstrated differential expressions levels between patients with non-severe and severe leptospirosis. In the validated cohort of circulating miRNAs, qRT-PCR also illustrated that miR-155-5p and miR-630 were both upregulated in the severe leptospirosis group. To our knowledge, this is the one of the first study to explore the role of circulating microtransriptome profiles in severe leptospirosis. Among the six differentially expressed miRNAs that were evaluated, miR-155-5p had the strongest association with the severity of leptospirosis with an AUC of 0.63 (95%CI;0.513–0.748, p = 0.023). In the stepwise analysis, miR-155-5p expression was a better prognostic indicator when combined with HCO_3-_ levels with an AUC of 0.79 (95%CI;0.692–0.893, p<0.001). Moreover, the addition of six serum miRNAs to the HCO3- level had the best discriminatory performance with a combined AUC of 0.82, 95%CI; 0.730–0.915, p<0.001) (Table 2).

Sepsis is defined as life-threatening organ dysfunction caused by a dysregulated host response to infection. Leptospirosis is one of the most common causes of sepsis, especially in the tropics [2], including Thailand [4]. Severe leptospirosis usually requires ICU admission, which can account for 30% of the hospitalised leptospirosis patients [5], where mortality in these patients may be as high as 40% [6]. In this study, 24% of the severe leptospirosis participants were admitted to ICU and the overall mortality rate of these severe patients was 21%. Thus, leptospirosis is still an important disease. It is necessary to develop a good predictor or biomarker in order to predict the outcome and monitor treatment response.

The miR-155-5p can regulate a large number of mRNA targets [27] and also is involved in various human biological processes, including the inflammatory response and immunity adaptation. In the inflammatory response, miR-155-5p transcription can be activated by TLR stimulation, especially in macrophages and dendritic cells [28]. The upregulation of miR-155-5p also causes the release of pro-inflammatory cytokines, including interleukin (IL)-1β and tumour necrosis factor-α [28]. In adaptive immunity, miR-155-5p is upregulated in activated B and T lymphocytes and is involved in the differentiation of T-helper cells [29]. Accordingly, miR-155-5p knock out mice were found to have limited antibody production.

Macrophages play an essential role in leptospirosis by phagocytizing bacteria in humans [30]. Previous research suggests that *Leptospira* inside macrophages are fully capable of replication [31] and are capable of escaping host defense responses [32]. Transcriptomic analysis of J774A.1 macrophages after an 8-h infection with saprophytic, virulent, and attenuated *Leptospira spp*. revealed changes in the expression levels of 29 miRNAs from as early as 8 h [20], and so suggests they may have potential as markers for the early detection of inflammation. However, the miRNA expression profiles appeared to be dependent on the bacterial strain and attenuation, which was suggested to be due to the differences in the bacterial lipopolysaccharide and protein profiles. Only three miRNAs were associated with all treatments: 155-5p, 155-3p, and 221-5p. These could be universal predictors for *Leptospira spp*. infection and may not be related to severity of the disease. Without knowing the correlation between their expression levels, and the severity, and mortality of the disease, it does not allow the early and specific clinical treatment for life-threatening conditions. Our study was the first report to illustrate the differences in miRNA-155-5p expression levels between severe and non-severe patients. Src homology 2-containing inositol phosphatase-1 (SHIP-1), an inhibitor of inflammation, is a well-known target for miR-155-5p. Increased expression of miR-155-5p, with a resulting decrease of SHIP-1 and trigger the production of major pro-inflammatory cytokines, including TNF-α and IL-6 [33, 34]. We, therefore, hypothesize that SHIP-1 is downregulated by miR-155-5p, leading to the production of major pro-inflammatory cytokines, which might play a considerable role in the development of severe leptospirosis. We also found a good association between the upregulation of miRNA-155-5p and the mortality rate. As such, these finding suggest a potential biomarker for the early detection and prevention of sepsis development.

The expression of miR-155-5p in sepsis patients was found to be upregulated compared to in healthy subjects [35]. In addition, the circulating miR-155-5p levels correlated with the severity of disease and mortality; with and AUC for predicting mortality of 0.76. As in our study, serum miR-155-5p levels were higher in severe leptospirosis patients and the miR-155-5p expression level was upregulated in patients that subsequently died. The AUC for predicting mortality was 0.76, which is comparable to a previous study [35]. Previously, the association between long pentraxin PTX3, the protein that is in the same class as C-reactive protein, and mortality in severe leptospirosis was reported [36], where the serum PTX3 and IL-6 levels were higher in non-survivors. The accuracy of serum IL-6 and PTX3 levels for predicting mortality was acceptable, with an AUC of 0.75 and 0.70, respectively. Hence, the serum miR-155-5p and IL-6 are comparable in terms of predicting mortality.

In terms of miR-630, it has been reported to be deregulated and involved in tumor progression of many human cancer types [37–39], including renal cancer [40]. It was reported that miR-630 is upregulated in a majority of clear cell renal cell carcinoma patients, and its expression is an independent prognostic factor for patients with renal cancer [40]. A recent study indicated that miR-630 function as oncotherapy-obstructing microRNAs by directly targeting organic cation transporter 2 in renal cell carcinoma [41]. However, the regulation of miR-630 in infectious diseases has not yet been reported before.

This study had several strengths. First, we used three standard laboratory confirmatory tests to diagnose leptospirosis: direct culture, MAT, and PCR. Second, we collected the samples on the first day of the visit for biomarker testing, which is more practical than collecting the samples at the first day of symptoms. Third, in term of sample hemolysis, which has been reported to alter miRNA measurements in whole blood, plasma, serum, and tissues [42–44]. We have been identified the effect of hemolysis in serum and found that average ratio of indicator miR-23A to miR-451 below seven. Therefore, the serum samples might not have been affected by hemolysis. Fourth, we used the NanoString nCounter gene expression system to discover the candidate miRNAs. This technique requires small amount of sample but offers comprehensive quantitative miRNA profile with high sensitivity and specificity. Although this technique is quite expensive, it is less complicated than microarray and RNA-seq. approaches. Subsequently, we confirmed the selected miRNA expression with qRT-PCR. However, to our knowledge, there is no similar study to support our findings on this matter. We, therefore, encourage further study to explore the performance of this biomarker.

There are some limitations in our study. We tested the biomarker on the first day of visit, not on the first day when the symptoms were present. Severe leptospirosis participants in our study had already developed clinical manifestations of organ failure. Hence, we can only determine the association of miRNA with disease severity and not for prediction. In addition, we measured the miRNA level only once on the first day of enrolment and not when the patient's condition had changed or after intervention had been applied. Nonetheless, biomarkers are more useful as a prognosis predictor if they are measured earlier. This study is a prospective observational study that used the stored serum samples from the leptospirosis confirmed participants and serum samples are not available in most cases, which disallowed us to determine the possible effect of the studied miRNAs on some immunologic markers such as cytokines and immune cell populations. Moreover, there are several challenges to overcome in order to determine the accurate level of expression of miRNA in serum and to ensure that both the positive and negative results are reliable. First, miRNAs detected in serum samples may have a cellular origin and may be relevant in terms of biomarker discovery. It is important to avoid cellular contamination and hemolysis. Second, the serum sample contains very low amounts of RNA. Thus, precautions should be taken to prevent RNase contamination and degradation of the RNA sample.

In conclusion, miR-155-5p was upregulated in severe leptospirosis patients and it's used as a predictor of the clinical outcome remains to be tested. Furthermore, the prognostic characteristics were even better when the miR-155-5p expression level was combined a clinical parameter, such as the bicarbonate level. A large prospective study is suggested to confirm the usefulness of its applications.

## Supporting information

S1 Fig Relative expression of circulating microRNAs in patients with leptospirosis and healthy donors. (TIF)

S2 Fig Boxplots with individual variables of circulating microRNAs in patients with leptospirosis. (TIF)

S3 Fig Correlations of serum miRNA levels, disease severity and markers of inflammation. (TIF)

S1 File NanoString miRNA profiling data file. (XLSX)

S2 File The qRT PCR raw data file. (XLSX)
