# Supplementary figures and images for: Circulating microtranscriptome profiles reveal distinct expression of microRNAs in severe leptospirosis
Source: PLoS Negl Trop Dis. 2020 Nov 11;14(11):e0008809. doi: 10.1371/journal.pntd.0008809 (PMC7682886; doi:10.1371/journal.pntd.0008809)

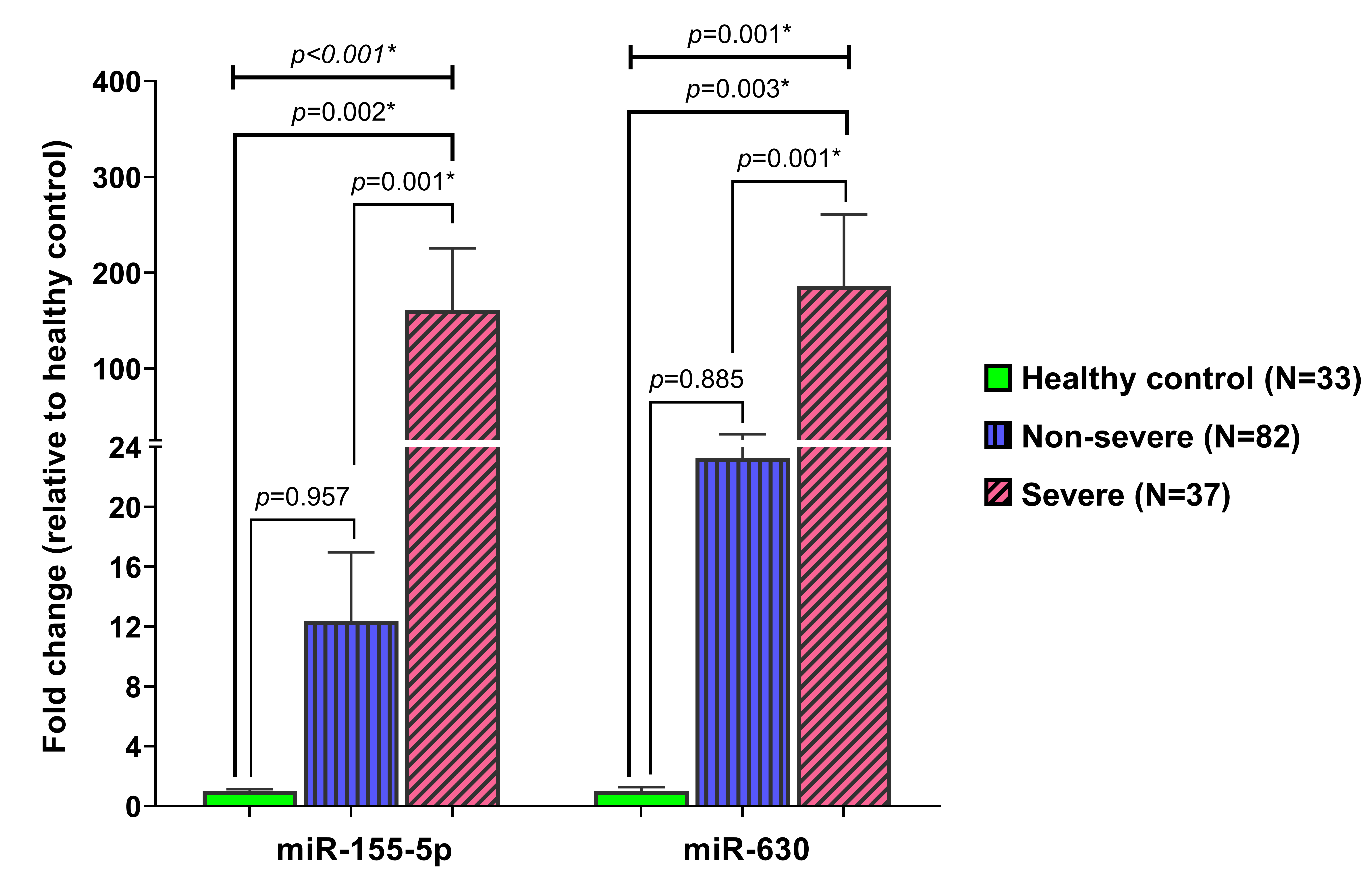

Supplement: S1 Fig — (TIF) [file pntd.0008809.s001.tif]

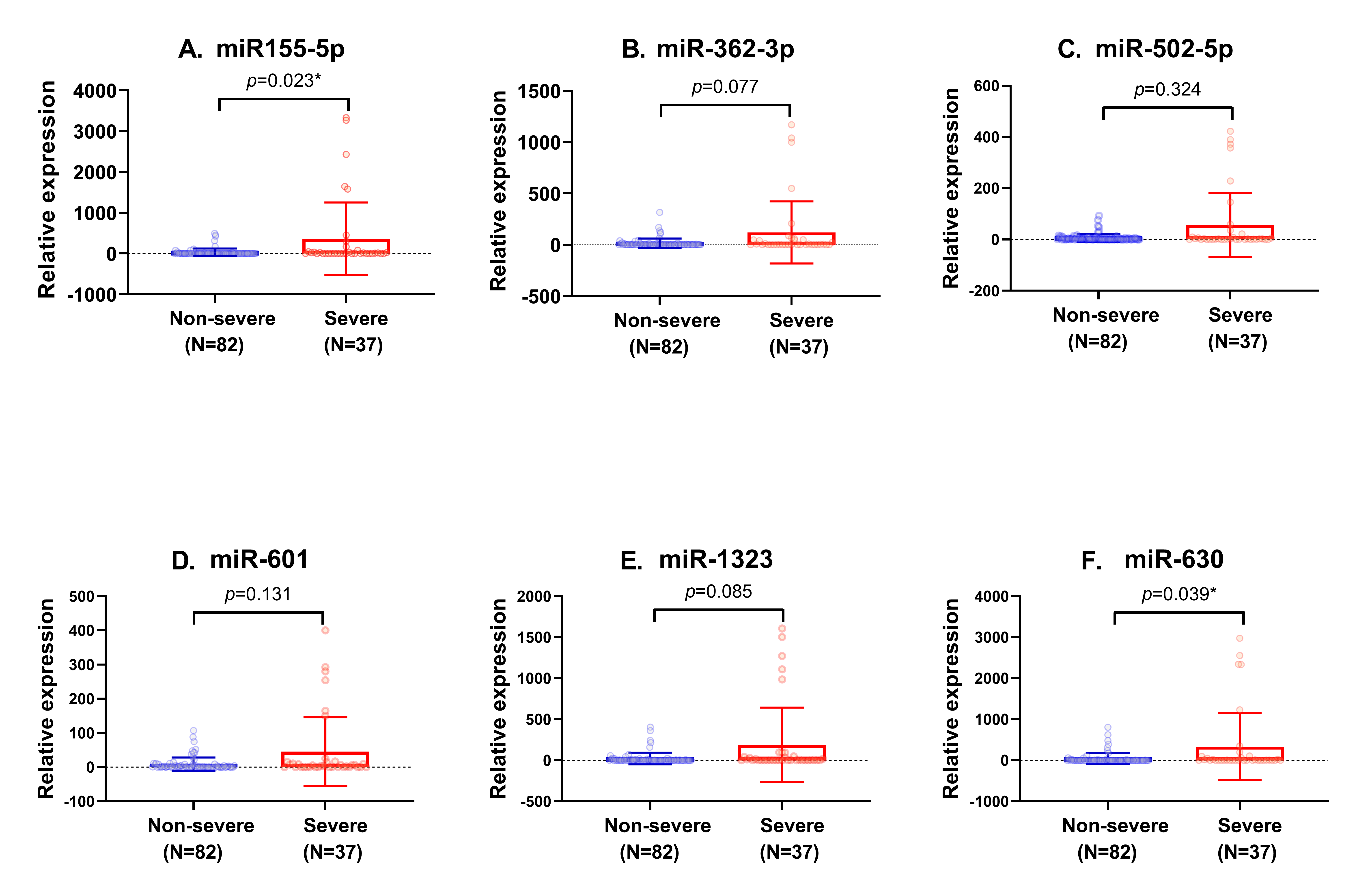

Supplement: S2 Fig — (TIF) [file pntd.0008809.s002.tif]

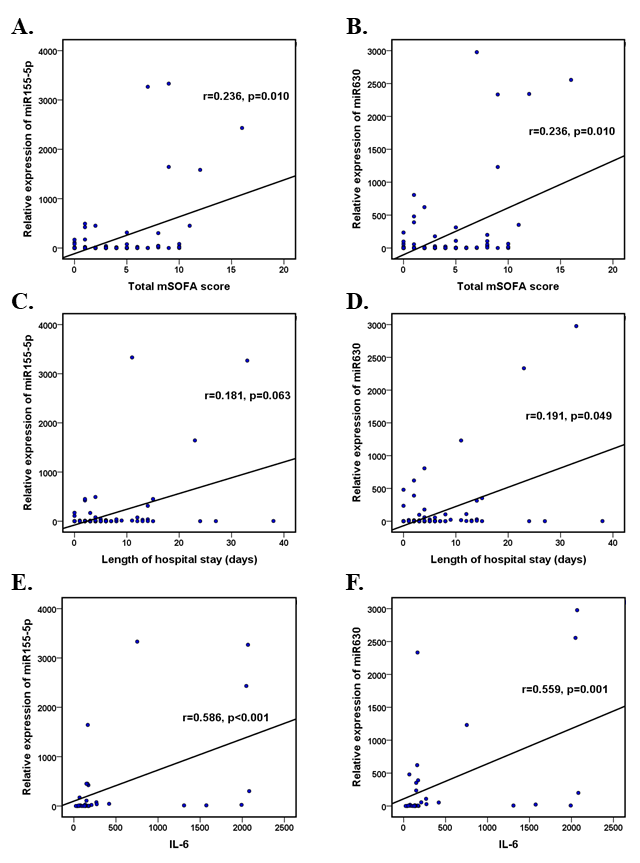

Supplement: S3 Fig — (TIF) [file pntd.0008809.s003.tif]
